# The effect of consignment to broodmare Sales on physiological stress measured by faecal glucocorticoid metabolites in pregnant Thoroughbred mares

**DOI:** 10.1186/1746-6148-10-25

**Published:** 2014-01-17

**Authors:** Martin Schulman, Annet Becker, Stefanie Ganswindt, Alan Guthrie, Tom Stout, Andre Ganswindt

**Affiliations:** 1 Section of Reproduction, Faculty of Veterinary Science, University of Pretoria, Private Bag XO4, Onderstepoort 0110, South Africa; 2 Summerhill Stud, PO Box 430, Mooi River 3300, South Africa; 3 Department of Anatomy and Physiology, Faculty of Veterinary Science, University of Pretoria, Private Bag XO4, Onderstepoort 0110, South Africa; 4 Equine Research Centre, Faculty of Veterinary Science, University of Pretoria, Private Bag XO4, Onderstepoort 0110, South Africa; 5 Department of Equine Sciences, Faculty of Veterinary Medicine, Utrecht University, Yalelaan 114, 3584 CM Utrecht, The Netherlands; 6 Mammal Research Institute, Department of Zoology and Entomology, University of Pretoria, Pretoria 0002, South Africa

## Abstract

**Background:**

Validation of a method for the minimally-invasive measurement of physiological stress will help understanding of risk factors that may contribute to stress-associated events including recrudescence of Equid herpesvirus (EHV), which is anecdotally associated with sales consignment of pregnant Thoroughbred mares. In this study we compared two similar groups of late-gestation Thoroughbred broodmares on the same farm: a consigned Sales group (N = 8) and a non-consigned Control group (N = 6). The Sales mares were separated from their paddock companions and grouped prior to their preparation for, transport to, and return from the sales venue. Both groups were monitored by sampling at regular intervals from 5 days prior to until 14 days after the sales date (D0) to measure physiological stress in terms of changes in faecal glucocorticoid metabolite (FGM) concentrations, and for event-related viral recrudescence via daily body temperature measurements and periodic nasal swabs for PCR analysis for EHV-1 and -4 DNA.

**Results:**

In both groups, FGM levels increased post-sales before returning to pre-sales levels. Specifically, FGM concentrations in the Sales mares were significantly higher on D + 3 and D + 10 than on D-4 and D-3 (F = 12.03, P < 0.0001, Post hoc: P = 0.0003 – 0.0008) and in the Control group FGM concentrations were higher on D + 10 than D-4 (F = 5.52, P = 0.004, Post hoc: P = 0.005). Interestingly, mean FGM levels in Control mares were significantly higher at 4 of the 5 sampling points (t = 5.64 – 2.25, p = 0.0001 – 0.044). Only one (Sales) mare showed PCR evidence of EHV-1 shedding.

**Conclusions:**

Using FGM to measure physiological stress was supported by the increases observed in all mares after Sales consignment, including those not consigned to the sale. Monitoring FGM levels therefore represents an appropriate, minimally-invasive method for future studies to assess the contribution of physiological stress to EHV recrudescence in horses transported to sales or equestrian events.

## Background

The physiological stress response in horses involves various metabolic, immunological and neuro-endocrine mechanisms [1-5]. Moreover, chronic exposure to stress may result in immunosuppression and an increased susceptibility to disease [1,5]. The interpretation of serum cortisol levels, which are commonly employed as an indicator of stress in horses [3-5], is however complicated by the effects of episodic fluctuations and pulsatile secretion of this hormone [1,2,4,6-8]. To avoid obtaining non-representative samples, assessment of adrenocortical function can be standardized using faeces as a hormone matrix for measuring time-averaged response to a stressor [9-11]. Monitoring changes in faecal glucocorticoid metabolite (FGM) output offers several advantages in that faeces are easily collected with minimal handling of the animal and the FGM concentrations measured reflect cumulative secretion and elimination of hormones over several hours [8]. Enzyme immunoassays (EIA) have been developed and validated for monitoring changes in FGM output in various species, including the horse [9,12]. The lag time between stress-related plasma hormone release and the associated appearance of the signal in faeces is approximately 24–48 h [4] and the changes in FGM concentrations do not appear to be significantly influenced by changes in grass or other food intake [2]. Faecal FGM measurement therefore represents a practical, non-invasive method to monitor adrenocortical endocrine function in horses, overcoming potential shortcomings of serum cortisol assays.

It has been suggested that one of the key events in the pathogenesis of Equid herpesvirus (EHV) abortion is stress-associated reactivation of latent infection followed by transfer and replication of the virus in the upper respiratory epithelium and local lymph nodes. Virus is subsequently shed via nasal secretions, while cell-associated viraemia also occurs [13,14]. In pregnant mares, transfer of abortogenic virus to the placenta, resulting in placentitis and abortion has been reported [15-17]. Stress-associated reactivation of latent EHV-1 is one proposal advanced for why EHV-1 abortion is still seen, albeit, sporadically in vaccinated broodmare groups [13,17,18]. The risk factors for viral recrudescence (including potential stressors) are however poorly understood [19]. The few reported studies investigating potential relationships between specific stress factors and viral reactivation and shedding are limited by the absence of any data concurrently verifying the physiological effects of the presumed stressor [19,20]. Relocations, including sales’ consignment and the introduction of new mares into established groups of pregnant Thoroughbred broodmares are anecdotally associated with subsequent EHV-associated sequelae including abortion. In such cases, it is assumed that the relevant potential stressors include social, environmental and management-associated cues [13,17,18].

The primary aim of this study was to investigate changes in faecal glucocorticoid metabolite (FGM) measurements during the course of the events associated with routine sales consignment of pregnant broodmares. Secondarily, event-related viral reactivation was monitored concurrently by daily monitoring of body temperature and molecular detection of the presence of viral nucleic acid for EHV by real-time quantitative PCR [14,21] in samples recovered from the upper respiratory tract by nasal swabbing.

## Methods

The study was approved by the University of Pretoria’s Animal Use and Care Committee (V068/05).

### Animals

Twenty-six Thoroughbred broodmares of ≥ 7 months gestation, aged from 3–14 years and resident on a farm in KwaZulu-Natal Province, South Africa were included in this study during the southern hemisphere winter of 2010. The mares were distributed among routinely-managed groups of approximately 30 pregnant mares, grouped based on anticipated foaling dates. Mares were kept permanently outdoors in paddocks of 4.6-11.7 ha in area that provided free access to kikuyu grass (*Pennisetum clandestinum*) pasture. All mares were routinely vaccinated with an inactivated EHV-1 vaccine at 5, 7 and 9 months of gestation. Eight mares were destined for consignment at an annual sale of broodmares and designated as the ‘Sales group’. Six mares, of a similar stage of pregnancy but not destined for sales consignment were selected as the Control group. Twelve additional mares were monitored to increase the number of data points for analyzing the relationship between FGM levels and body temperature.

### Study design

The Sales mares were removed from their original respective groups approximately one month prior to the sales and transferred to a common, separate paddock within sight and sound of their original paddock group-mates. The key events identified as potential stressors in Sales mares were: temporary removal for pregnancy confirmation by trans-rectal palpation and ultrasound five days prior to the sale (D-5), washing and grooming two days prior to the sale (D-2), transportation by road to an unfamiliar sales venue 30 km away with a journey time of approximately one hour on the morning of the sale (D0), being stabled individually at the venue prior to and after the short duration of the auction process in the sales ring, returning to the farm on the same day *via* the manner and route described above, and upon return being maintained as a single, separately managed group as a biosecurity precaution until foaling.

### Sample collection and analyses

The broodmares were all accustomed to routine, frequent gynaecological examinations, including trans-rectal palpation, and were monitored using minimal manual restraint within their paddocks between D-5 to D + 14 by daily recording of rectal temperature and recovery of a faecal sample. Faecal samples were collected daily between 08 h00 and 10 h00 by manual extraction using a lubricated latex-gloved hand inserted *per* rectum. The glove with homogenized faecal material inside was tied, labeled with an indelible marker pen and removed for immediate storage at –20°C until hormone extraction and assay.

In addition nasal secretions were collected by swabbing at five points: D-4, -3, +3, +10 and +14. The nasal secretions, obtained by insertion of a sterile cotton-tipped swab^a^*via* the *external nares*, were placed in a pre-labeled plastic sleeve and stored at -20°C prior to molecular detection of viral nucleic acid using qPCR.

#### Faecal extraction and hormone analysis

Frozen faecal samples were lyophilized, pulverized, and sifted using a metal mesh strainer to remove fibrous material [22]. Approximately 0.05 g of the faecal powder was then extracted by vortexing for 15 min with 80% ethanol in water (3 ml). Following centrifugation for 10 min at 1500 g, supernatants were transferred into micro-centrifuge tubes and stored at -20°C until analysis. Faecal extracts were measured for immunoreactive FGM concentrations using an EIA that detects 11, 17 dioxoandrostanes, and previously validated for monitoring adrenocortical endocrine function in a range of mammals including horses [12,23,24]. Serial dilutions of faecal extracts gave displacement curves which were parallel to the standard curve of the assay. Sensitivity of the assay at 90% binding was 3.0 pg/well. Intra- and Inter-assay coefficients of variation, determined by repeated measurements of high- and low-value quality controls, ranged between 5.2% and 13.7%. The cross-reactivities of the antibody and the assays performed on microtiter plates were previously reported [25,26].

#### PCR for EHV

Nasal swabs were agitated for 5 s in 0.5 ml of 0.1 M PBS (pH 7.4) in a 1.5 ml Eppendorf tube. Nucleic acid was extracted from 100 μl of the preparation using MagMAX™ Pathogen DNA/RNA kit^a^ and a Kingfisher 96 Magnetic Particle Processor^b^ according to the manufacturer’s protocol. Subsequently, a duplex PCR for EHV-1 and EHV-4 was performed using previously described primers and probes [27]. Briefly, 17 μl of a mastermix consisting of 1 μl of each primer/probe mix, 5 μl of nuclease free water and 10 μl of Kapa Probe Fast ABI Prism® 2X PCR master mix^c^ was added to each well of a PCR plate and 3 μl of the extracted template was added. Positive and negative template controls were included on each plate. The PCR was performed according to the manufacturer’s protocol with the assignment of a cut-off value of < 40 cycle s (Ct) for positive detection of viral DNA.

### Data and statistical analyses

Differences in FGM levels between two sets of data were examined by t-test, after confirming between-group equivalence of variances. Differences in hormone concentrations between more than two sets of data were examined by one-way repeated measures ANOVA, followed by post hoc analysis using Tukey’s test. All tests were two-tailed, with significance set at *P* ≤ 0.05. In cases of all-pairwise multiple comparison procedures, the α-level was adjusted by applying a previously described procedure [28]. The relationship between the two variables (FGM levels and body temperature) was examined using Pearson’s product–moment correlation test. Statistical analysis was performed using SigmaPlot 12^d^.

## Results

**Figure 1:**
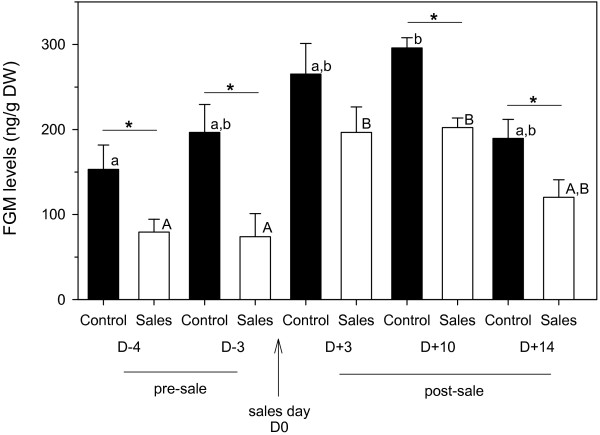
**FGM concentrations (mean + SEM) of mares in the control (N = 6) and sales groups (N = 8) at the selected sampling points D-4, D-3, D + 3, D + 10 and D + 14 with respect to the day of the sales.** Asterisks indicate statistically significant differences between the two groups at a respective sampling point, determined using t-tests. Different superscripts (capital letters = Sales group; lower case letters = Control group) indicate statistically significant differences between sampling points within the respective group, determined using one-way repeated measures ANOVA.

FGM levels of both Sales and Control mares showed an overall increasing trend of between the pre-sales period and the initial post-sales period, before returning to pre-sales levels again (Figure 1). For the Sales group, FGM levels were significantly lower on both D-4 and D-3 when compared to respective FGM concentrations on D + 3 and D + 10 (F = 12.03, P < 0.00001, power of performed test = 0.999, α = 0.05, Post hoc: P = 0.0003 – 0.0008). A significant increase in FGM concentrations between D-4 and D + 10 was also found for the Control group (F = 5.52, P = 0.004, power of performed test = 0.899, α = 0.05, Post hoc: P = 0.005). Somewhat unexpectedly, the mean FGM concentrations for Control mares were higher than in Sales mares, with a significant difference between the two groups at 4 of the 5 of the individual sampling points (t = 5.64 – 2.25, p = 0.0001 – 0.044).

**Figure 2:**
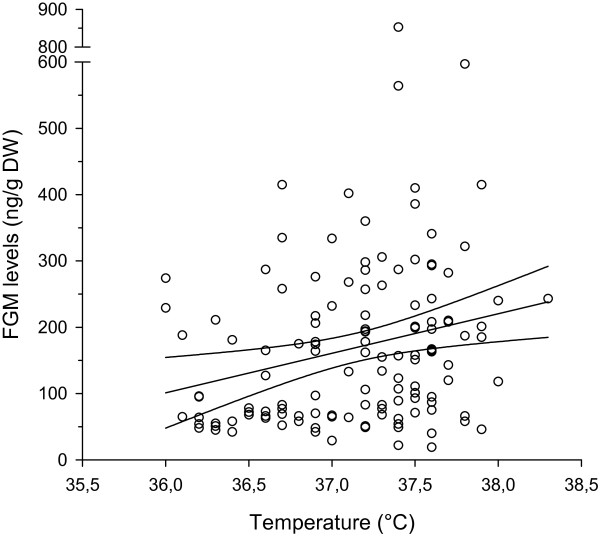
Relationship between FGM levels and body temperature (r = 0.238, n = 130, p = 0.006) determined in 26 pregnant Thoroughbred broodmares of > 7 months gestation, monitored up to seven times over a selected 19 day pre- to post-sale period.

Only two of the daily recordings of body temperatures exceeded the lower threshold defined to indicate pyrexia, namely ≥ 38.0°C, with one incident in each of two Sales mares. In addition, only one incident of viral shedding of EHV-1 was detected by PCR during the course of this study (Ct = 34.7), and this in a Sales mare on D + 14. Over the complete range of data points, FGM levels and rectal temperatures were weakly but significantly correlated (r =0.238, n = 130, p = 0.006) (Figure 2).

## Discussion

The utility of FGM levels as a means of monitoring stress associated with sales consignment, or indeed other potential stressors, among pregnant broodmares was supported by the current data. The major stressful events (i.e. transport to, individual stabling and sales’ ring appearance, and return from the sales) on day 0 were associated with a significant rise in FGM levels 3 days later, which would account for the expected lag-time of 24–48 h before the respective changes in glucocorticoid concentrations would appear in the faeces [1,4]. An interesting, if slightly unexpected, finding was that mares that were not consigned for sale showed significantly higher pre-sales FGM concentrations than the Sales mares at nearly all time points, and a rise in FGM concentrations in the days after their herd mates had been taken to the sales, that reached statistical significance on D + 10. Although it is difficult to separate the individual stressors that contributed to the observed response, this suggests that social disruption (in this case removal of Sales mares from the settled groups and subsequent removal from visual and olfactory contact for approximately 24 h) may be a key stressful event among settled groups of horses; this echoes a previous study investigating the effects of social instability on chronic stress [29]. The raised FGM concentrations in non-consigned (control) mares may have been influenced by a “neighbour” stress effect, given that the consigned mares remained within sight and sound of their previous group mates on all except the day of sales. Alternatively or additionally, it is probable that removal of an animal from a group disrupted the social order and resulted in a potential stress-related period during which a new hierarchical equilibrium (‘pecking order’) was established. The comparatively high FGM levels in non-consigned mares subsequent to disruption of their group stability indicate that the paradigm of increased abortion-risk in pregnant mares as a result of social stress should also include consideration of the susceptibility of mares in a group from which others have been removed. It is, however, less easy to interpret why the Sales mares did not show higher FGM levels in the pre-sales period as a result of removal from their group and introduction into a new, albeit smaller group.

A weak but statistically significant association between body temperature and FGM levels was observed but is difficult to interpret, not only because the incremental rises in FGM with increasing body temperature are modest but also because of the potentially confounding effect of some of the stressful events (e.g. transportation) which could result in mild hyperthermia concomitant with stress. Future observations in larger populations may clarify whether there is a biological association between FGM and body temperature, or whether the correlation detected represents parallel increases (stress and hyperthermia) induced by a specific stressful event. The present study’s limited success in associating stressful events with clinical pyrexia and associated EHV shedding was similar to previous studies [19,21]. This was not particularly surprising and most probably related to the small sample population, the relatively short sampling period, sampling frequency, the effects of vaccination and, not least that stress-induced reactivation of latent EHV infection is almost certainly an uncommon event.

## Conclusions

This study described a novel approach to quantify physiological stress in Thoroughbred broodmares during late gestation after their exposure to potential stressors during a series of events anecdotally-associated to predispose to EHV recrudescence and associated abortion. The study provided useful preliminary data to support the value of FGM monitoring as a minimally-invasive but reliable method for monitoring stress when investigating EHV reactivation, or indeed other events proposed to be a result of medium to long-term stress, in horse populations.

## Endnotes

^a^Life Technologies™, Carlsbad, CA, USA.

^b^Thermo Fisher Scientific Inc., Waltham, MA, USA.

^c^Kapa Biosystems, Cape Town, South Africa.

^d^Systat Software Inc., San Jose, CA, USA.

## Abbreviations

ANOVA: Analysis of variance; DNA: Deoxyribose-nucleic acid; EHV: Equid herpesvirus; EHV-1: Equine herpesvirus type 1; EIA: Enzyme immune-assay; FGM: Faecal glucocorticoid metabolite; PBS: Phosphate-buffered saline; qPCR: Quantitative real-time polymerase chain reaction; RNA: Ribose-nucleic acid.

## Competing interests

The authors declare they have no competing interests.

## Authors’ contributions

MS conceived and participated in the design of the study, organised and participated in the data collection and interpretation and drafted the manuscript. AB participated in the data collection and study execution. SG performed the faecal extraction and EIA. AGuthrie participated in the design of the study, participated in the data collection and interpretation and in the PCR analysis. TS participated in data interpretation and drafting of the manuscript. AGanswindt participated in the study execution, interpretation, draft of the manuscript and performed the statistical analysis. All authors read and approved the final manuscript.
